# Supplementary figures and images for: Integrated Transcriptome and Metabolome Analysis Elucidates the Defense Mechanisms of Pumpkin Against Gummy Stem Blight
Source: Int J Mol Sci. 2025 Mar 13;26(6):2586. doi: 10.3390/ijms26062586 (PMC11941995; doi:10.3390/ijms26062586)

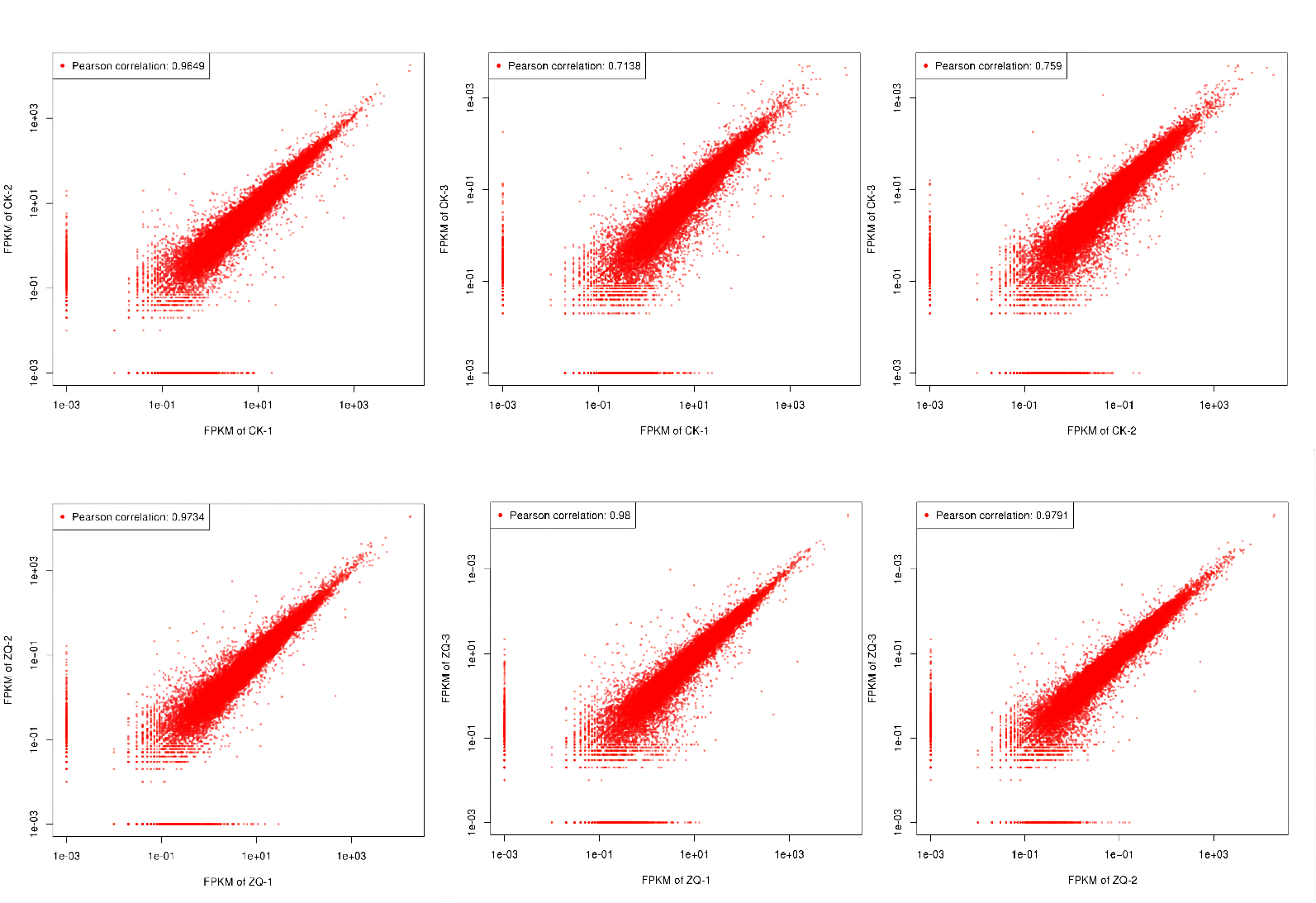

Supplement: Supplementary file 1 [file ijms-26-02586-s001.zip › Figure S1.png]

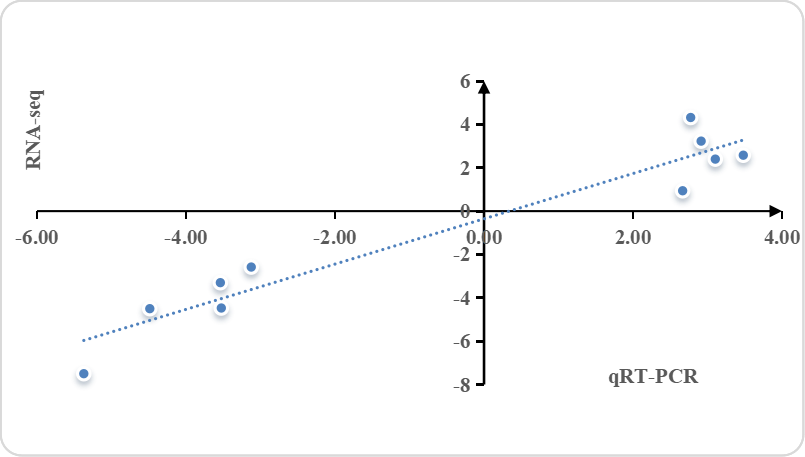

Supplement: Supplementary file 1 [file ijms-26-02586-s001.zip › Figure S2.png]

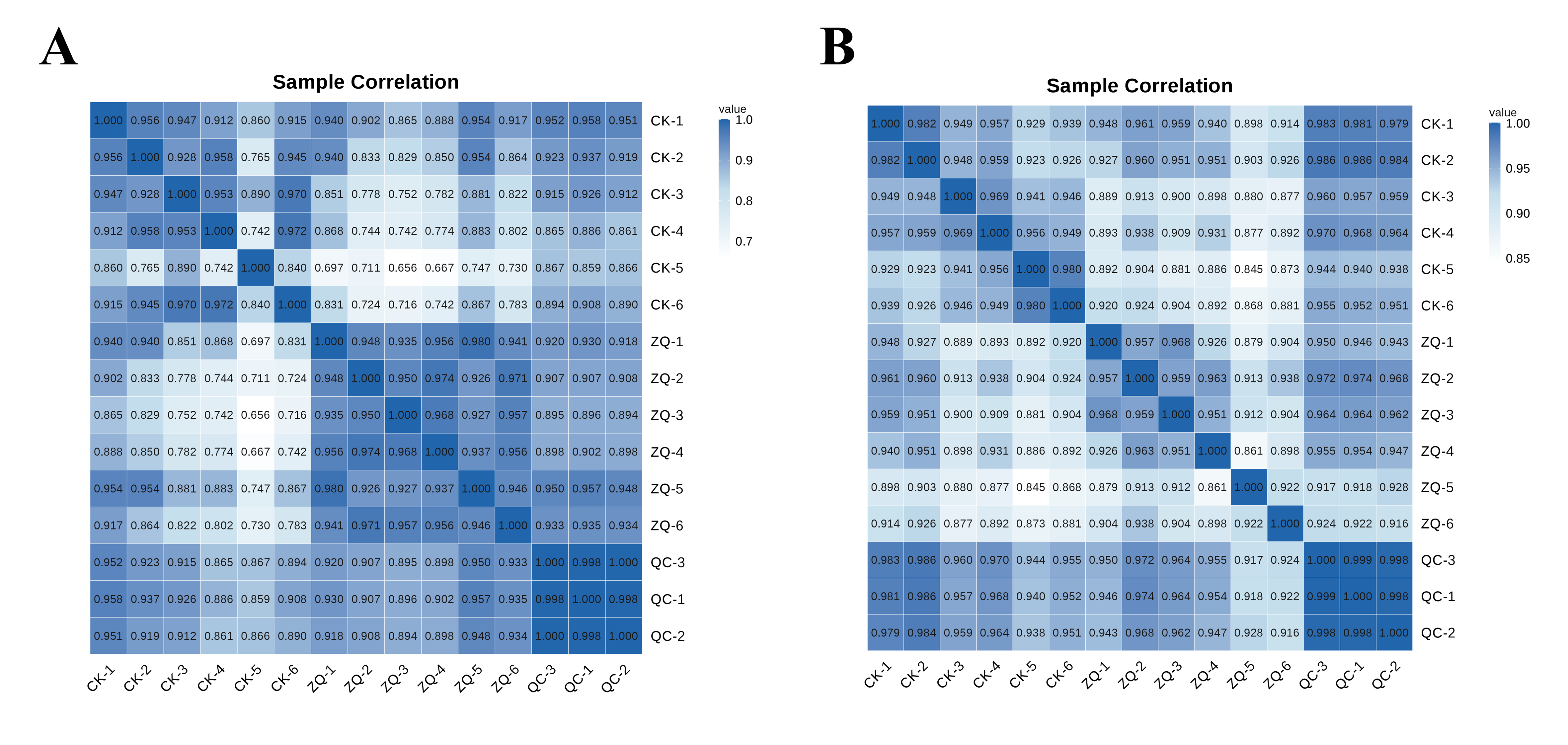

Supplement: Supplementary file 1 [file ijms-26-02586-s001.zip › Figure S3.tif]

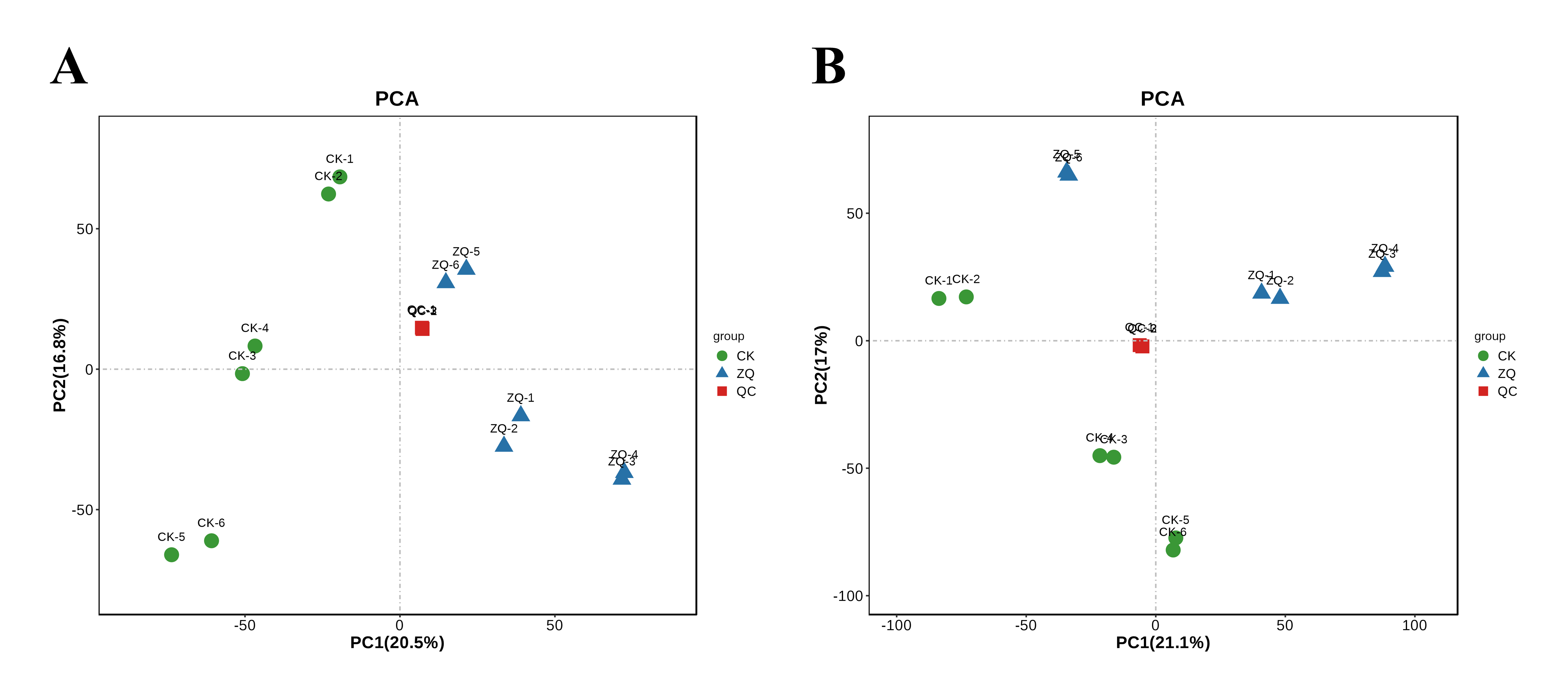

Supplement: Supplementary file 1 [file ijms-26-02586-s001.zip › Figure S4.tif]

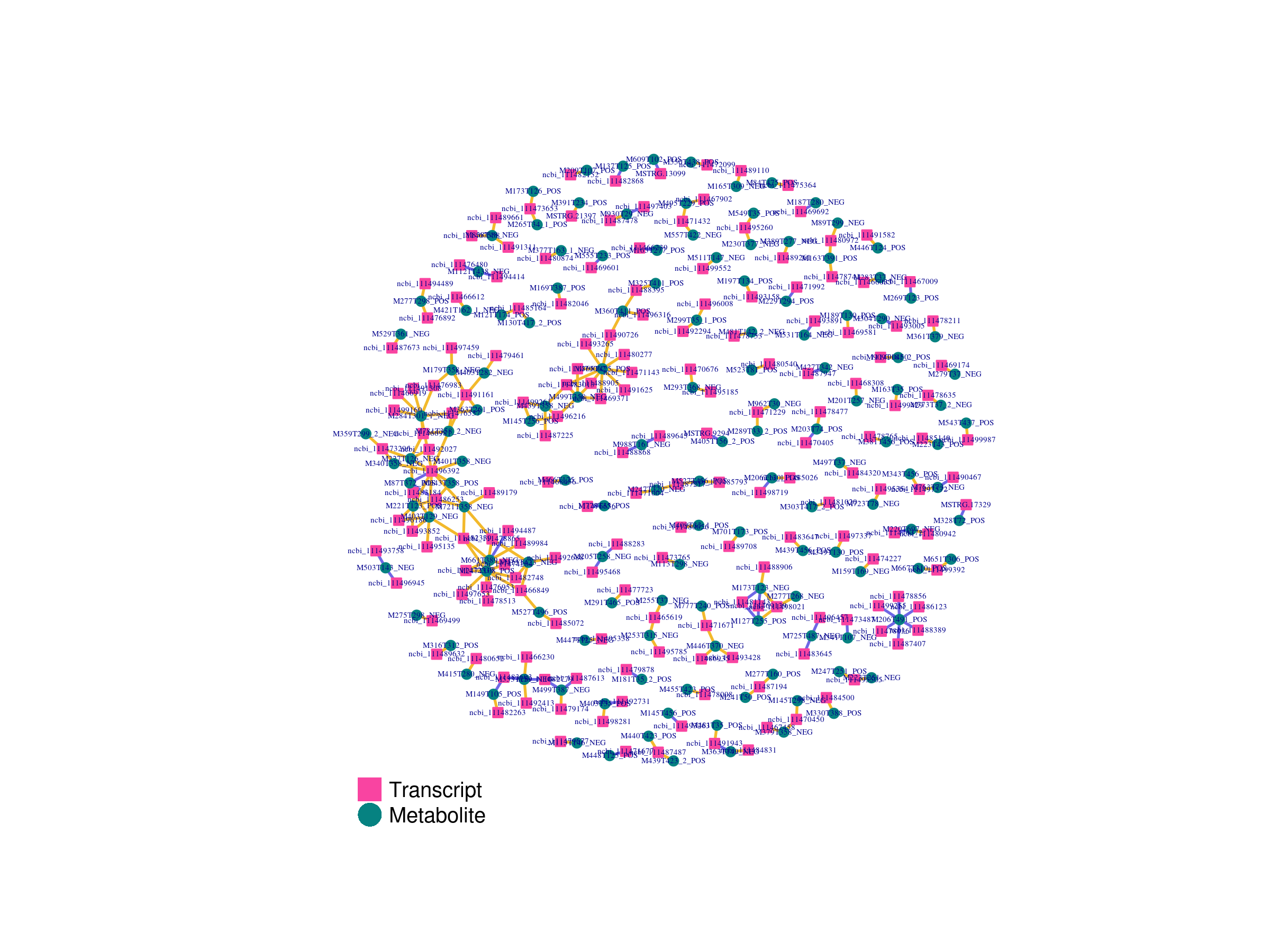

Supplement: Supplementary file 1 [file ijms-26-02586-s001.zip › Figure S5.png]
